# A machine learning approach to predicting inpatient mortality among pediatric acute gastroenteritis patients in Kenya

**DOI:** 10.1002/lrh2.10478

**Published:** 2024-12-26

**Authors:** Billy Ogwel, Vincent H. Mzazi, Bryan O. Nyawanda, Gabriel Otieno, Kirkby D. Tickell, Richard Omore

**Affiliations:** ^1^ Kenya Medical Research Institute‐Center for Global Health Research (KEMRI‐CGHR) Kisumu Kenya; ^2^ Department of Information Systems University of South Africa Pretoria South Africa; ^3^ Department of Computing United States International University Nairobi Kenya; ^4^ Department of Global Health University of Washington Seattle Washington USA

**Keywords:** acute gastroenteritis, diarrhea, machine learning, mortality, prediction

## Abstract

**Background:**

Mortality prediction scores for children admitted with diarrhea are unavailable, early identification of at‐risk patients for proper management remains a challenge. This study utilizes machine learning (ML) to develop a highly sensitive model for timelier identification of at‐risk children admitted with acute gastroenteritis (AGE) for better management.

**Methods:**

We used seven ML algorithms to build prognostic models for the prediction of mortality using de‐identified data collected from children aged <5 years hospitalized with AGE at Siaya County Referral Hospital (SCRH), Kenya, between 2010 through 2020. Potential predictors included demographic, medical history, and clinical examination data collected at admission to hospital. We conducted split‐sampling and employed tenfold cross‐validation in the model development. We evaluated the sensitivity, specificity, positive predictive value (PPV), negative predictive value (NPV), and the area under the curve (AUC) for each of the models.

**Results:**

During the study period, 12 546 children aged <5 years admitted at SCRH were enrolled in the inpatient disease surveillance, of whom 2271 (18.1%) had AGE and 164 (7.2%) subsequently died. The following features were identified as predictors of mortality in decreasing order: AVPU scale, Vesikari score, dehydration, sunken eyes, skin pinch, maximum number of vomits, unconsciousness, wasting, vomiting, pulse, fever, sunken fontanelle, restless, nasal flaring, diarrhea days, stridor, <90% oxygen saturation, chest indrawing, malaria, and stunting. The sensitivity ranged from 46.3%–78.0% across models, while the specificity and AUC ranged from 71.7% to 78.7% and 56.5%–82.6%, respectively. The random forest model emerged as the champion model achieving 78.0%, 76.6%, 20.6%, 97.8%, and 82.6% for sensitivity, specificity, PPV, NPV, and AUC, respectively.

**Conclusions:**

This study demonstrates promising predictive performance of the proposed algorithm for identifying patients at risk of mortality in resource‐limited settings. However, further validation in real‐world clinical settings is needed to assess its feasibility and potential impact on patient outcomes.

## 1 INTRODUCTION

Despite the global decline in diarrheal mortality, diarrheal disease is still the third leading cause of death in children <5 years, killing approximately 443 832 children annually.^1^, ^2^, ^3^ Majority of these deaths occur in low‐to‐middle‐income countries (LMICs).^4^ The risk of death among children with moderate to severe diarrhea is 8.5 times higher compared to their counterparts who are healthy.^4^ Some of the leading causes of diarrheal deaths in the past have been severe dehydration and fluid loss but more recently septic bacterial infections has emerged as an important etiology of all diarrhea‐associated mortality.^5^ Additionally, malnutrition and impaired immunity increases risk of life‐threatening diarrhea.

While dehydration scores,^6^ severity scores,^7^ and diarrhea case management guidelines informed by the Integrated Management of Childhood illness guidelines^8^ are available in hospitals, mortality prediction scores for children admitted with diarrhea are unavailable. Additionally, demanding work environments, burnout, and the pressure of attending to severely ill patients may reduce healthcare providers' ability to systematically assess risk.^9^, ^10^, ^11^ In light of these challenges, development of tools which can support clinician judgment in early identification and management of at‐risk patients is important. Machine learning (ML) has been adopted in medical practice to rapidly develop data‐driven prediction models for various clinical questions.^12^, ^13^, ^14^ Machine learning has been successfully used to develop mortality prediction models for various diseases, including COVID‐19^15^; Acute Myocardial Infarction^16^; Chagas disease^17^; Cancer.^18^ However, no such predictive models exist in literature for diarrhea‐associated mortality.^19^

The aim of this study was to build and evaluate the performance metrics of various ML models in the prediction of mortality using data collected from children aged <5 years hospitalized with diarrhea at Siaya County Referral Hospital (SCRH).

## 2 QUESTION(S) OF INTEREST

1. What variables are associated with diarrhea‐associated inpatient mortality?
2. Which machine learning algorithm is able to develop a highly sensitive tool for identifying children admitted with diarrhea at increased risk of inpatient mortality?

## 3 METHODS

### 3.1 Study site and population

We utilized de‐identified demographic, clinical, anthropometric measurements, physical examination, and outcome data collected during rotavirus surveillance by the Kenya Medical Research Institute‐Center for Global Health Research at SCRH. This study enrolled pediatric patients hospitalized with acute gastroenteritis (AGE)—defined as ≥3 looser than normal stools and/or ≥1 episode of unexplained vomiting followed by loose stool within a 24‐h period beginning within the 7 days before seeking healthcare. The study site and population have been previously described.^20^, ^21^ In summary, SCRH has a pediatric bed capacity of 60 and serves a culturally homogenous and predominantly rural population. The study area is malaria endemic^21^ with a high HIV prevalence (15.3%)^22^ and under five mortality ratio (159/1000 live births).^23^ The nurse and doctor to population ratios are 1:1697 and 1:38 511, respectively.^23^ This study focused on children aged <5 years hospitalized with AGE at SCRH from January 2010 through December 2020.

### 3.2 Statistical analysis

We compared clinical and demographic characteristics of deaths versus live discharges among children admitted with AGE aged <5 years. Proportions were reported for categorical variables and either chi‐square or Fisher's exact test was performed as appropriate. Student's *t*‐test and Wilcoxon rank sum tests were used to compare continuous variables as appropriate. We used Cochran–Armitage trend test to assess the trend of death over time. We calculated the sample size for the study using the formula developed by Riley et al.^24^ and got a sample size of at least 2122 observations. The details of the sample size calculation are included in the Supplementary material.

### 3.3 Modeling

Our study followed the guidelines for developing and reporting ML predictive models in biomedical research.^25^ We employed seven ML algorithms in building models to predict the likelihood of AGE associated death among children aged <5 years. The algorithms used included: Logistic Regression (LR), Naive Bayes (NB), Random Forest (RF), Gradient Boosting (GBM), Support Vector Machine (SVM), K‐Nearest Neighbors (KNN), and Artificial Neural Networks (ANN).

In the model development process, missing data points in the predictor variables were handled using the Multiple Imputation by Chained Equations (MICE) package,^26^ which allows one to impute data and includes several functions for identifying the missing data patterns present in a dataset. The overall missingness map and patterns in missing data are shown in Figure S1, respectively. The Boruta package,^27^ an all relevant feature selection wrapper algorithm that selects relevant features by comparing original attributes' importance with importance achievable at random, estimated using their permuted copies, was then used to implement feature selection in order to improve model accuracy, reduce computational cost, and improve interpretability of the model. We assessed for correlation among selected variables using Cramer's V statistic and found that sunken eyes and skin turgor were highly correlated with dehydration. However, we opted to keep all selected variables to maintain high model sensitivity since excluding them caused an 18% drop in model sensitivity. The dataset was partitioned into training and test data in the ratio of 75%:25%.^28^

The mortality predictive models were developed in the training dataset using tenfold cross‐validation^29^, ^30^ to avoid under‐fitting or over‐fitting of the model, and subsampling techniques^31^ were employed within the resampling procedure to handle class imbalance in our outcome variable (mortality) since a disparity in the frequencies of the observed classes can have a significant negative impact on model fitting. The models from the training data were evaluated in the test dataset using the following performance metrics: sensitivity, specificity, positive predictive value (PPV), negative predictive value (NPV). The models were then optimized using hyper‐parameter tuning.^32^ Receiver operating characteristic (ROC) curves were constructed and the area under the curve (AUC) for each model was computed. The champion model was the best predictive model from the pool of seven algorithms used based on the outlined performance metrics. Given the gravity outcome variable (mortality), we prioritized sensitivity in our cutoff criteria to ensure that high‐risk cases are accurately identified. To determine the optimal cutoff point, we plotted the ROC curve and used Youden's index to select the threshold that maximizes sensitivity. We also assessed the predictive performance of existing diarrhea scores, including dehydration score, Vesikari score, and IMCI general danger signs, to determine their potential utility in predicting diarrhea‐associated mortality.

Calibration which involves comparing the model's prediction against the real (observed) distribution was assessed using Brier scores, Spiegelhalter's *z*‐test and its accompanying *p*‐value.^33^ We calibrated the champion model using Platt scaling approach in which model estimates are transformed by passing them through a trained sigmoid function.^33^ We also developed a custom stacked ensemble model to assess if it improved performance. We conducted explanatory model analysis (EMA) for the top models (AUC >80.0%) using a model agnostic procedure to estimate Shapley additive explanations (SHAPs) attributions. This was implemented using the DALEX package.^34^ The SHAP values were plotted as bar plots in descending degree of importance with the red color signifying a negative association and green color showing a positive association.

The modelplotr package^35^ was used to generate evaluation plots (cumulative gains, cumulative lift, response, and cumulative response) to assess the business value of the champion model. These plots provide insights into the model's performance in terms of targeting the desired outcome (target class), including its ability to identify high‐value cases and its relative effectiveness compared to random selection. Descriptive analysis, predictive modeling for mortality, and plotting were all performed in R version 4.1.2.^36^

### 3.4 Ethical consideration

The study protocol was approved by the KEMRI Scientific and Ethical Review Unit (SERU# 1801) and Institutional Review Board of CDC (CDC IRB #3308). Caregivers provided written informed consent before initiation of study procedures. Furthermore, this work utilized de‐identified data. Additionally, ethical approval for undertaking the current study was sought from the health research ethics committee of the University of South Africa, College of Agricultural Sciences (2023/CAES_HREC/2192).

## 4 RESULTS

**FIGURE 1:**
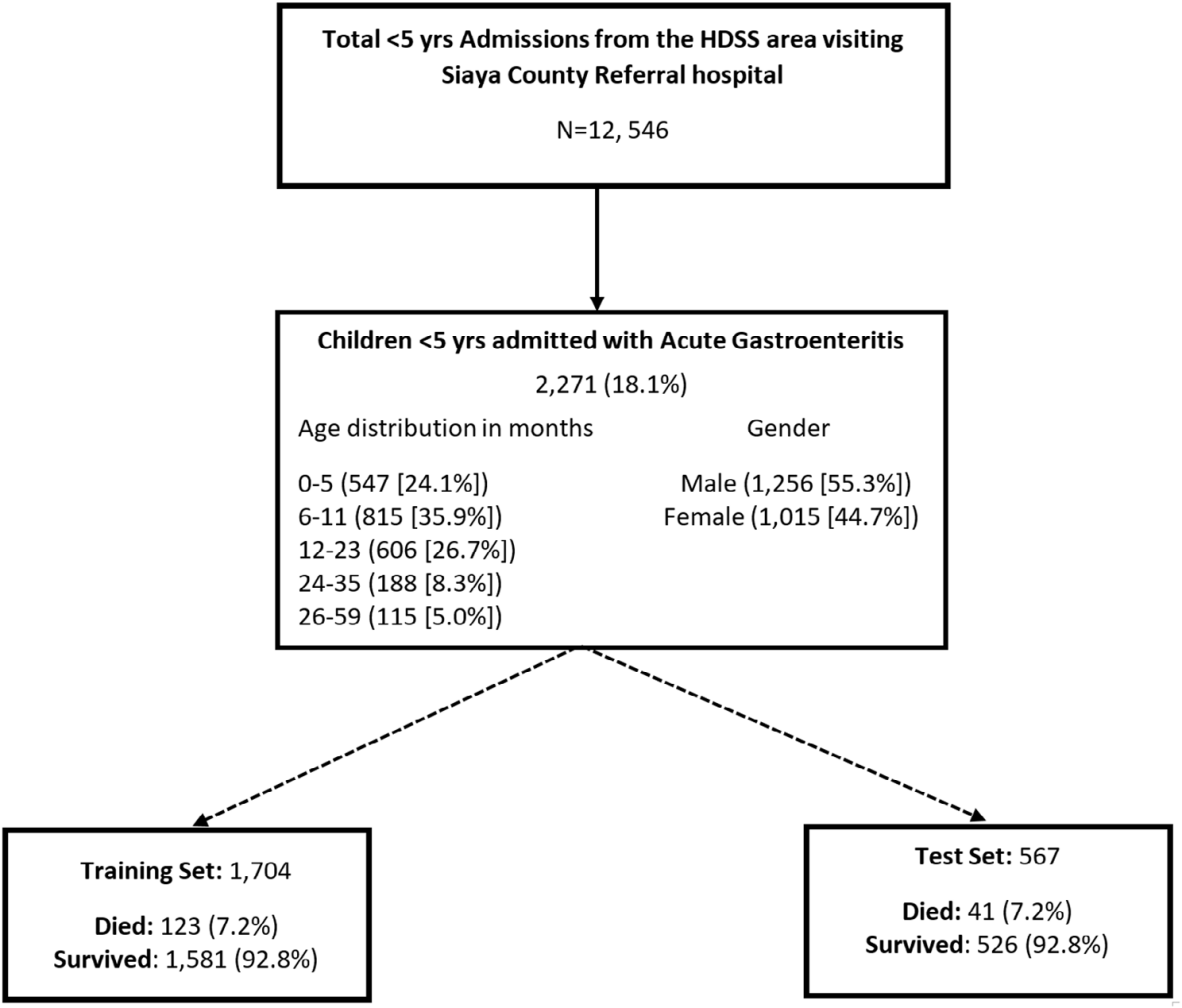
Flow diagram of train/test splitting of data and deaths among children aged <5 years hospitalized for acute gastroenteritis at Siaya County Referral Hospital, western Kenya 2010–2020.

During the study period, 12 546 children aged <5 years admitted at SCRH were enrolled in the inpatient disease surveillance, of whom 2271 (18.1%) met criteria for AGE. AGE patients had a median age of 9.9 months with an interquartile range of (6.1–17.4). The gender distribution was 1256 (55.3%) and 1015 (44.7%) for males and females, respectively. Among the 2271 with AGE, 164 (7.2%) died. The median (IQR) days between admission and death was 2 (1–4) days. The prevalence of death among AGE cases did not vary significantly over the years (*p* = 0.3142), it was highest in 2013 (*n* = 24/167 [12.6%]) and lowest in 2018 (*n* = 0/47 [0.0%]) (Table S1). The enrollment flowchart and the train/test splitting of the dataset is shown in Figure 1.

The characteristics of patients stratified by mortality status are shown in Table 1. There was no significant difference in age between children who died and those who survived (median age in months [IQR]: 10.4 [5.9–18.2] vs. 9.9 [6.1–17.3], *p* = 0.9723). Compared with those who survived, those who died had a lower oxygen saturation (median [IQR]: 96 [91–98] vs. 97 [94–98], *p* = 0.0013), a higher number of diarrhea days (median [IQR]: 4 [3–6] vs. 3 [2–4], *p* < 0.0001), and a lower temperature (median [IQR]: 37.0 [36.0–38.0] vs. 37.2 [36.6–38.0], *p* = 0.0037). Additionally, vomiting, unconsciousness, weight loss, Alert, Verbal, Pain, and Unresponsive (AVPU) scale, restless, sunken eyes, skin pinch, capillary refill, drinks poorly, visible wasting, dehydration, stunting, wasting, chest indrawing, stridor, and nasal flaring were significantly associated with death. The overall HIV prevalence in our study was 9.2% (99/1079). However, compared to those who survived, the HIV prevalence among those who died did not differ significantly (9/71 [12.7%] vs. 90/1008 [8.9%], *p* = 0.290).

**TABLE 1:** Characteristics of patients aged <5 years admitted at Siaya County Referral Hospital with acute gastroenteritis: 2010–2020.

Characteristics	Died (*n* = 164)	Survived (*n* = 2107)	*p*‐value
Median age months[IQR]	10.35 [5.865–18.185]	9.86 [6.11–17.28]	0.9723
Age			
0–5 months	43 (26.2)	504 (23.9)	0.177
6–11 months	50 (30.5)	765 (36.3)	
12–23 months	53 (32.3)	553 (26.3)	
24–35 months	14 (8.5)	174 (8.3)	
36–59 months	4 (2.4)	111 (5.3)	
Gender: Male	85 (51.8)	1171 (55.6)	0.353
Vital signs			
Median Temperature[IQR]	37 [36–38]	37.2 [36.6–38]	**0.0037**
Median Oxygen Saturation[IQR]	96 [91–98]	97 [94–98]	**0.0013**
Median Respiratory Rate [IQR]	41 [32–52]	42 [34–52]	0.5648
Symptoms			
Vomiting	56 (34.4)	505 (24.0)	**0.003**
Convulsions	29 (18.5)	87 (19.0)	0.938
Lethargic	85 (59.9)	949 (50.9)	0.119
Unconscious	41 (26.6)	211 (10.4)	**<0.0001**
Fever	114 (72.6)	1786 (87.4)	**<0.0001**
Malaria	23 (15.7)	683 (35.0)	**<0.0001**
Lost weight	115 (78.2)	1166 (62.0)	**0.001**
Unable to drink	10 (8.7)	78 (5.5)	0.234
AVPU scale			
Alert	130 (81.3)	2032 (97.1)	**<0.0001**
Voice	18 (11.3)	37 (1.8)	
Pain	7 (4.4)	20 (1.0)	
Unresponsive	5 (3.1)	3 (0.1)	
Restless	54 (33.3)	480 (22.9)	**<0.0001**
Convulsing Now	2 (1.2)	92 (1.4)	0.863
Sunken Eyes	72 (43.9)	567 (27.1)	**<0.0001**
Skin Pinch			
Normal	69 (42.1)	1460 (69.7)	**<0.0001**
Slowly	80 (48.8)	534 (25.5)	
Very Slowly	15 (9.1)	102 (4.9)	
Capillary Refill (>2 s)	35 (22.3)	314 (15.4)	**0.024**
Drink			
Not able to drink/drinks poorly	90 (55.6)	703 (33.8)	**<0.0001**
Drinks eagerly/Very Thirsty	48 (29.6)	866 (41.7)	
Normally	24 (14.8)	509 (24.5)	
Bulging Fontanelle	5 (4.0)	24 (1.6)	**0.049**
Sunken Fontanelle	40 (27.4)	332 (18.2)	**0.007**
Red Eyes	2 (1.6)	5 (0.3)	**0.035**
Visible wasting	38 (31.7)	93 (6.4)	**<0.0001**
IVF given	94 (57.7)	817 (39.2)	**<0.0001**
Dehydration status			
Severe dehydration	23 (14.0)	117 (5.6)	**<0.0001**
Some dehydration	76 (46.3)	647 (30.7)	
No dehydration	65 (39.6)	1343 (63.7)	
Vesikari score			
Mild	25 (15.2)	294 (14.0)	0.389
Moderate	56 (34.2)	834 (39.6)	
Severe	83 (50.6)	979 (46.4)	
HIV positive	9 (12.7)	918 (91.1)	0.290
Stunting			
Normal	72 (44.7)	1361 (65.1)	**<0.0001**
Moderate	22 (13.7)	345 (16.5)	
Severe	67 (41.6)	384 (18.4)	
Wasting			
Normal	73 (45.9)	1450 (70.6)	**<0.0001**
Moderate	22 (13.8)	256 (12.4)	
Severe	64 (40.3)	349 (17.0)	
Underweight			
Normal	50 (30.7)	1320 (63.9)	**<0.0001**
Moderate	26 (16.0)	342 (16.5)	
Severe	87 (53.4)	405 (19.6)	
Prior Admission	6 (5.1)	29 (2.1)	**0.041**
Sought Care	109 (75.2)	1106 (60.7)	**0.001**
Chest indrawing	54 (34.2)	325 (16.0)	**<0.0001**
Stridor	16 (10.8)	84 (4.5)	**0.001**
Nasal flaring	94 (33.1)	346 (28.5)	**<0.0001**
Median Diarrhea days [IQR]	4 [3–6]	3 [2–4]	**<0.0001**
Median Diarrhea episodes [IQR]	4 [3–5]	4 [3–5]	0.6514
Median Vomit episodes [IQR]	2 [1–4]	3 [1–4]	**0.0143**
Median Vesikari Score [IQR]	11 [8–13]	10 [8–12]	0.1392

*Note*: Significance level: *p* < 0.05 (in bold).

Abbreviations: AVPU, alert, verbal, pain, and unresponsive scale; IQR, interquartile range; IVF, intravenous fluid.

**FIGURE 2:**
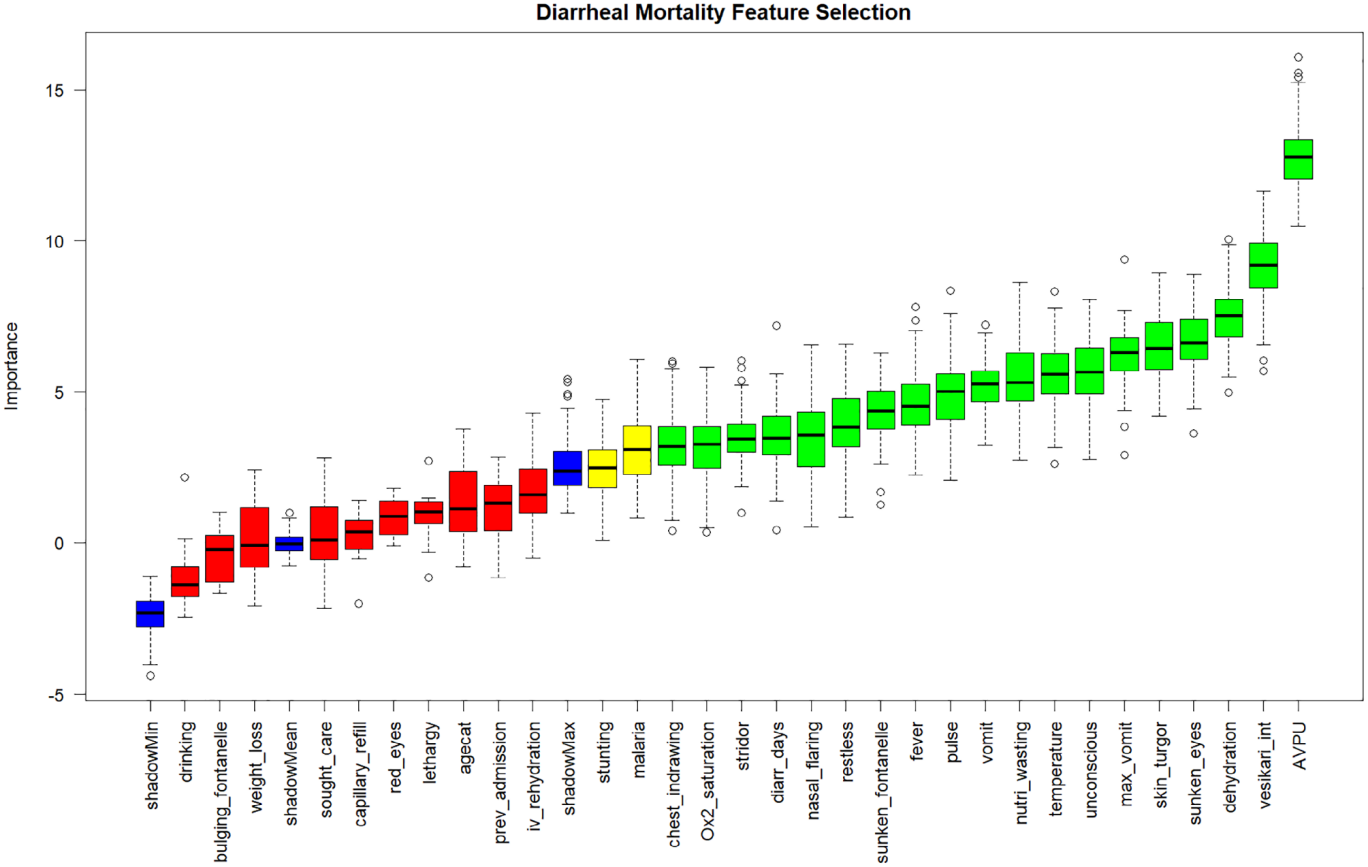
Feature selection for mortality among children aged <5 years hospitalized for acute gastroenteritis at Siaya County Referral Hospital, western Kenya 2010–2020.

From the feature selection analysis, the confirmed variables in order of importance were AVPU scale, Vesikari score, dehydration, sunken eyes, skin pinch, maximum number of vomits, unconsciousness, wasting, vomiting, pulse, fever, sunken fontanelle, restless, nasal flaring, diarrhea days, stridor, <90% oxygen saturation, and chest indrawing. While malaria and stunting were tentative variables (Figure 2).

**FIGURE 3:**
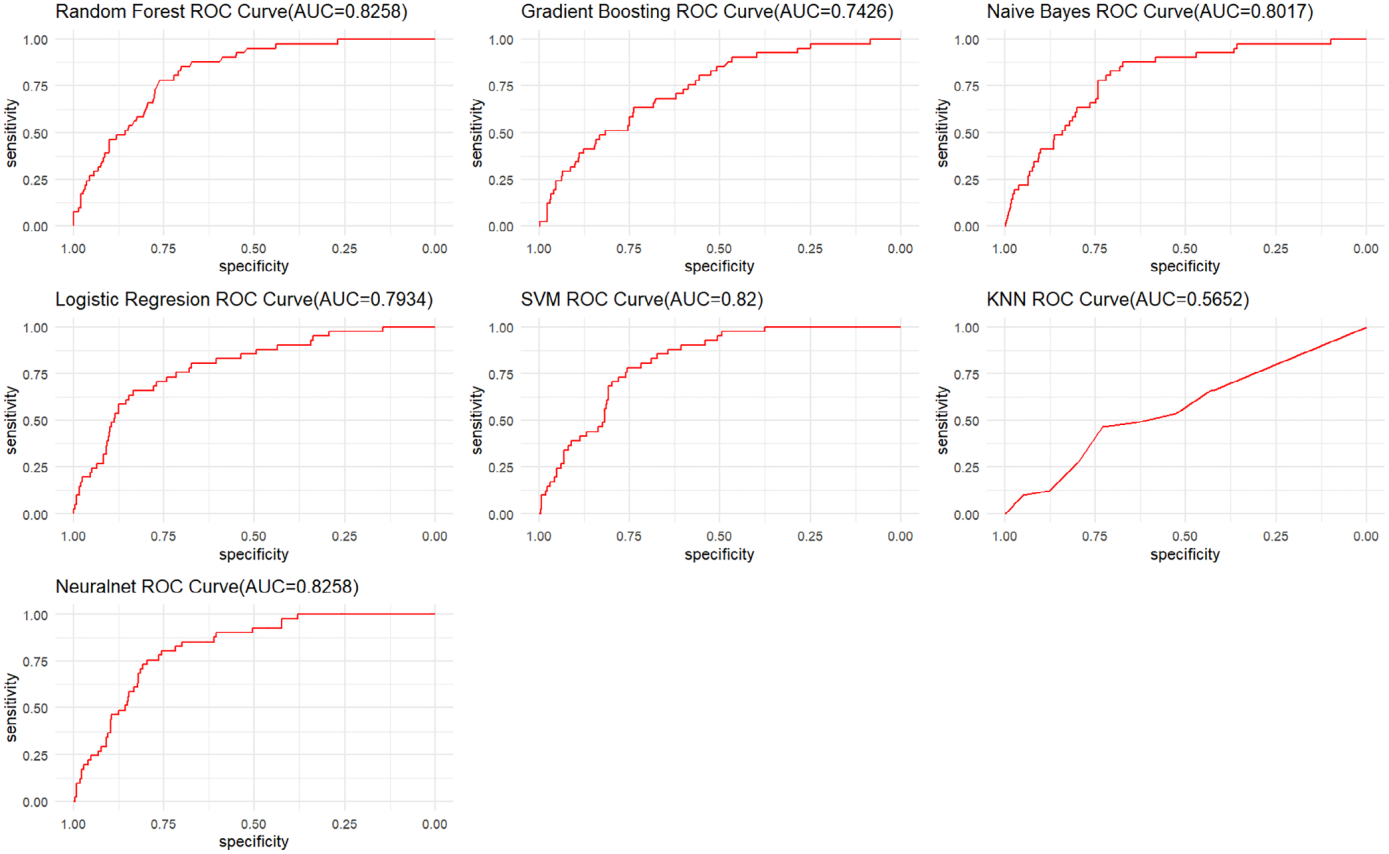
Receiver operator characteristic (ROC) chart for various machine learning algorithms predicting in‐hospital mortality among children aged <5 years admitted at Siaya County Referral Hospital, western Kenya 2010–2020.

We evaluated seven ML algorithms in the prediction of mortality among children admitted with AGE. From the models built using the confirmed and tentative variables, sensitivity was highest in the RF and NB models (78.0%) followed by the ANN model (75.6%), LR (73.2%), GBM (61.3%), and lowest in the KNN model (46.3%). The specificity ranged from 71.7% to 78.7%. Specifically, the specificity of the ANN model was the highest at 78.7%, followed by RF (76.6%), SVM (74.1%), GBM (74.0%), NB (73.4%), KNN (72.8%), and lowest in the LR model at 71.7%. The PPV ranged between 11.0% and 21.7% while the NPV ranged between 94.6% and 97.8%. The AUC of the models in decreasing order was 82.6%, 82.0%, 80.2%, 79.3%, 74.3%, and 56.5% for RF and ANN, SVM, NB, LR, GBM, and KNN, respectively (Table 2). The ROC curves for prediction models are shown in Figure 3.

**TABLE 2:** Mortality prediction models for patients aged <5 years admitted with acute gastroenteritis at Siaya county referral hospital, western Kenya 2010–2020.

Algorithm	Mortality				
	Sensitivity % [95% CI]	Specificity % [95% CI]	PPV % [95% CI]	NPV % [95% CI]	AUC % [95% CI]
RF	78.0 [62.4–89.4]	76.6 [72.8–80.2]	20.6 [14.6–27.9]	97.8 [95.9–99.0]	82.6 [77.1–88.1]
GBM	61.0 [44.5–75.8]	74.0 [70.0–77.0]	15.4 [10.2–21.9]	96.0 [93.7–97.7]	74.3 [66.9–81.7]
NB	78.0 [62.4–89.4]	73.4 [69.4–77.1]	18.6 [13.11–25.2]	97.7 [95.7–99.0]	80.2 [73.8–86.5]
LR	73.2 [57.1–85.8]	71.7 [67.6–75.5]	16.8 [11.6–23.1]	97.2 [95.0–98.6]	79.3 [72.3–86.4]
SVM	78.0 [62.4–89.4]	74.1 [70.2–77.8]	19.0 [13.4–25.8]	97.7 [95.8–99.0]	82.0 [76.8–87.2]
KNN	46.3 [30.7–62.6]	72.8 [68.8–76.6]	11.7 [7.2–17.7]	94.6 [91.9–96.6]	56.5 [47.4–65.7]
ANN	75.6 [59.7–87.6]	78.7 (75.0–82.1)	21.7 (15.2–29.3]	97.6 [95.7–98.9]	82.6 [77.2–88.0]

Abbreviations: 95% CI, 95% confidence interval; ANN, artificial neural networks; AUC, Aarea under the curve; GBM, gradient boosting machine; KNN, K‐nearest neighbors; LR, logistic regression; NB, naïve bayes; NPV, Nnegative predictive value; PPV, Ppositive predictive value; RF, random forest; SVM, support vector machine.

**FIGURE 4:**
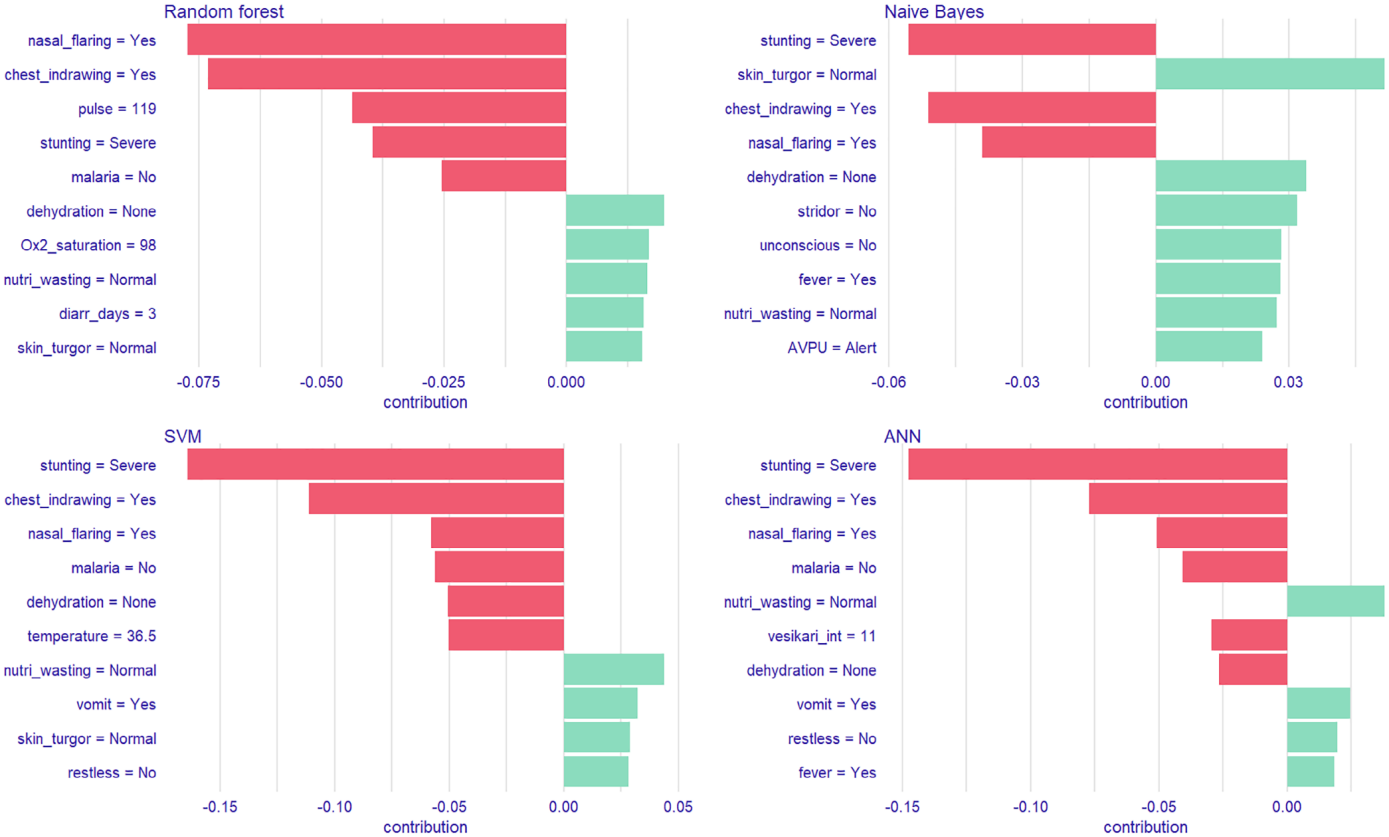
Explanatory model analysis for top four mortality predictive models.

The performance of the stacked ensemble model was 80.5%, 73.6%, 19.2%, and 98.0% for sensitivity, specificity, PPV, and NPV, respectively. The RF and ANN model had the best AUC model. We optimized the champion model using Youden's index to improve overall model accuracy, the optimal cutoff point above which the predicted probability would be classified as a case was determined to be 44.5%. The model achieved a sensitivity of 85.4% (95% CI: 70.8–94.4) and a specificity of 70.2% (95% CI: 66.0–74.0). Furthermore, the evaluation of existing diarrhea scores in predicting mortality revealed suboptimal results: dehydration score (AUC: 67.9 [95% CI: 60.5–75.2]), Vesikari score (AUC: 50.3 [95% CI: 41.6–58.9]), and IMCI danger signs (AUC: 61.3 [95% CI: 53.2–69.4]). The Brier scores and Spiegelhalter's *z*‐score and *p*‐value for assessing model calibration are reported in Table S2. Overall, the Brier scores were low and ranged between 0.15–0.44, however, the Spiegelhalter's *p*‐value showed that five models (RF, NB, LR, KNN, and ANN) were not properly calibrated (*p* < 0.05). The calibrated RF model had the same sensitivity and NPV (97.8%) while there was a slight decline in specificity (75.9%), and PPV (20.1%). The EMA of the models with AUC >80.0% showed that chest indrawing, nasal flaring, stunting, and wasting decreased the risk of mortality in all the four models (RF, ANN, SVM, and MB), whereas malaria was the only variable that increased risk of death in all the four models (Figure 4). Additionally, Vesikari score and dehydration (ANN model) and pulse (SVM model) decreased the risk of mortality. Specifically for the champion model (RF), the SHAP attributions in descending order were chest indrawing (−0.08), nasal flaring (−0.07), wasting (0.03), stunting (−0.03), malaria negative result (−0.02), sunken eyes (0.02), dehydration (0.02), oxygen saturation (0.02), restless (0.01), and skin turgor (0.01).

Although the RF and ANN models had the same AUC, the ANN model performed poorly in business value evaluation. The RF model was able to identify 67% of deaths from the top 25% of cases based on model probabilities (cumulative gains plot) and also identify 2.8 times more deaths compared to random selection (cumulative lift plot) (Figure S2).

## 5 DISCUSSION

The adoption of digital technologies in the healthcare sector has given rise to diverse and complex data which is driving the development of data‐driven predictive models. This shift is further advanced by the benefits posed by such models to both the patient and the healthcare system. From our evaluation of the seven machine learning algorithms, RF model emerged as the champion model in predicting mortality among children admitted with AGE. Additionally, based on our feature selection, the variables identified as predictors of mortality can be categorized into five categories: diarrhea‐specific severity variables (Vesikari score, maximum number of vomits in last 24 h, vomiting, diarrhea days, fever, dehydration [skin pinch, restless, sunken fontanelle, and sunken eyes]); danger signs (unconsciousness, and AVPU scale); malnutrition (wasting and stunting); vital signs (<90% oxygen saturation and pulse); co‐infections (malaria and respiratory distress [chest indrawing, stridor, and nasal flaring]). The EMA identified the same variables as Boruta feature selection although the importance of the variables and direction of the associations were different. Specifically, the EMA results showed a negative association between chest indrawing, nasal flaring, Vesikari score, wasting and dehydration findings that are inconsistent with existing literature.

Broad variations were observed in the performance metrics of the mortality prediction from the ML prediction models. These variations can possibly be explained by the fact that different algorithms have different purposes and different approaches in the implementation of the task at hand (classification). These algorithms search for trends and patterns differently based on their family and purpose.^37^ Despite the variations in performance, the results show the potential use of these algorithms, especially the RF model, in the prediction of disease consistent with findings from other studies.^38^

The RF and ANN were the best performing models based on their AUC. However, the ANN performed poorly in the business value evaluation. Therefore, we settled on the RF model as the champion model. This RF model incorporates the 19 variables identified in the feature selection process, outlined in the results. Furthermore, the EMA results of this model showed that nasal flaring, chest indrawing, pulse, stunting, malaria, dehydration, <90% oxygen saturation, wasting, diarrhea days, and skin pinch were the most important variables in predicting mortality among children hospitalized for AGE, ranked in descending order of importance. The good performance can be explained by the RF algorithm's robust prediction power and its ability to mitigate over‐fitting.^39^ Its AUC was 82.6% which is considered excellent.^40^ The RF algorithm builds an ensemble of decision trees that are trained and the results aggregated using a bagging approach to increase overall results.^41^ Additionally, RF can model high dimensional data and use many trees in the ensemble. RF also estimates variable importance and has a method for handling class imbalance as well as estimate missing data.^41^ This finding is consistent with findings from other studies that have shown that RFs are superior to other supervised learning algorithms: Ooka et al. in the prediction of diabetes^42^; Lwendi et al. in the prediction of COVID‐19 severity and outcomes^43^; Lee et al. in a healthcare monitoring system.^44^ Furthermore, the RF model showed good business value as it was able to identify approximately three times higher number of deaths compared to a random selection if we picked the top 25% cases based on model probability, and it was able to select 67% of overall deaths from the same selection.

The KNN model was the worst in the prediction of mortality and this could possibly be due to the need for feature scaling, the difficulties arising from dealing with data with higher dimensions and its sensitivity to noise in the data as explained by Guo et al.^45^ In spite of ensemble models being useful in reducing variance and building more robust models, our stacked ensemble model did not have better performance compared to other individual models. Reduction in model interpretability of ensemble models further reduced its consideration as a champion model for this classification task.

The variables identified through feature selection in this study have been documented as risk factors for mortality.^46^, ^47^, ^48^, ^49^, ^50^, ^51^ The similarities of results with previous studies indicate that ML algorithms are robust, useful, and feasible to implement in the healthcare domain. Specifically, clinical indicators of severity such as dehydration and the Vesikari score are associated with an increased risk of patient mortality and are used to guide therapy.^5^, ^48^ Severe dehydration and fluid loss have been the leading causes of diarrheal deaths. Delays in care‐seeking by caretakers in LMICs exposes children to more severe manifestation of disease resulting in complications and death which could be averted.^49^ Malnutrition has also been shown to elevate risk of mortality among children with diarrhea due to weakened immune response, anorexia, decreased absorptive function, mucosal damage, and nutrient exhaustion.^47^, ^50^ Furthermore, comorbidities have been shown to aggravate disease severity and trigger mortality among children with diarrhea.^47^, ^51^ This could be explained by the possibility that comorbidities or the immune response to them may alter the gut flora thereby increasing vulnerability to or severity of intestinal infections.^51^ Lastly, general danger signs have been documented as signs of increased risk of mortality and are indicative of organ failure and immediate risk of death.^8^

Our EMA results across the top four models had common variables (chest indrawing, nasal flaring, stunting, and wasting) that decreased risk of mortality with only malaria shown to increase risk of mortality. This observed variations in SHAP attributions across models could be attributed to model architecture differences, model complexity, and differences in handling feature interactions. While the assessment of the EMA analysis of top models gives a holistic view of the predictors and their relationships with mortality, priority should be given to the interpretability and alignment of SHAP attribution with domain knowledge particularly when there is discordance among models. Specifically in our analysis, signs of respiratory distress, wasting, and stunting across all models, as well as dehydration and Vesikari score in ANN model were shown to decrease the risk of mortality. While these findings are inconsistent with existing literature that shows that severe disease and comorbidities increases the risk of mortality, we can hypothesize that healthcare workers consider severely ill children more vulnerable and in need of closer and meticulous monitoring as well as prompt management, as outlined in the World Health Organization's Emergency Triage Assessment and Treatment guideline for low resource settings, which would result in favorable outcomes, hence the negative association with mortality.^52^

Approximately seven of every 100 children admitted with AGE died during hospitalization. Our model has shown great potential in its ability to help clinicians identify children at an increased risk of death in a timely manner thereby initiating close monitoring and better management. This is particularly important in LMICs like our setting where diagnostic capabilities are scarce and healthcare worker‐to‐patient ratio is high leading to pressure and lethargy on the health workers which may reduce their clinical judgment and performance. Early identification cascaded by better management will possibly translate to reduced AGE‐associated mortality. This model could be deployed as web‐based applications using platforms such as R‐shiny or plumber,^53^, ^54^ or more ideally it could be integrated into electronic medical records systems^55^ ensuring it is aligned with the clinical workflow. Such simple and flexible deployment methodologies can allow rapid adoption of the model in clinical practice helping to complement clinician judgment in the timely identification of at‐risk patients.

Our study makes important contributions in predictive modeling of pediatric enteric and diarrheal disease research. However, our study has some limitations. The epidemiology and management of AGE have evolved over time, especially with the introduction of the rotavirus vaccine in Kenya in July 2014. Our data may not fully reflect these changes in AGE trends, and the reduced number of deaths post‐vaccine introduction limits our ability to stratify temporally. This highlights the need for continued monitoring and periodic retraining of the model as more data becomes available to ensure its continued accuracy and relevance. While AGE was a primary reason for the admission of these children, the lack of postmortem data makes it impossible to fully ascertain if AGE was the predominant cause of death. Furthermore, potential deaths occurring post‐discharge were not captured in the study, which could possibly lead to misclassification bias. Additionally, the study area has a high HIV prevalence, and while HIV is known to increase mortality risk, we did not observe a significant difference in mortality by HIV status, likely due to the high number of participants with missing HIV data. We did not conduct external validation to assess the robustness and generalizability of our model using new participant level data from individuals in potential populations. While we may be unable to quantify the generalizability of our model, the results from our model can be achieved in other settings with similar epidemiology of AGE. Future research could explore the independent validation of this model in a different population, and how clinical application of similar risk prediction tools alter patient care.

## 6 CONCLUSIONS

This study demonstrates promising predictive performance of the proposed algorithm for identifying children hospitalized for acute gastroenteritis at risk of mortality in resource‐limited settings. While the model shows strong predictive accuracy and shows promise in improving the early identification and management of at‐risk patients, further validation in real‐world clinical settings is needed to assess its feasibility and potential impact on patient outcomes.

## AUTHOR CONTRIBUTIONS

Billy Ogwel and Vincent H. Mzazi conceived the study; Billy Ogwel, Bryan O. Nyawanda, Gabriel Otieno, Vincent H. Mzazi, Kirkby D. Tickell, and Richard Omore contributed to study design and implementation; Billy Ogwel and Vincent H. Mzazi analyzed and interpreted the data. Billy Ogwel drafted the manuscript, and all authors critically reviewed the manuscript for intellectual content and approved the final manuscript. All authors read and approved the final manuscript.

## CONFLICT OF INTEREST STATEMENT

Authors declare no conflict of interest.

## Supporting information


**Figure S1.** (a) Missingness map for the mortality study for patients aged <5 years admitted with acute gastroenteritis at Siaya County Referral Hospital, western Kenya 2010–2020. (b) Patterns of missing data for the mortality study for patients aged <5 years admitted with acute gastroenteritis at Siaya County Referral Hospital, western Kenya 2010–2020.
**Figure S2.** Business value plots for the champion model predicting in‐hospital mortality among patients aged <5 years admitted with acute gastroenteritis at Siaya County Referral Hospital, western Kenya 2010–2020.
**Table S1.** Trends of mortality among patients aged <5 years admitted at Siaya County Referral Hospital with acute gastroenteritis: 2010–2020.
**Table S2.** Calibration results of mortality prediction models applied to test data.

## Data Availability

The data used for the modeling in this study belongs to KEMRI and restrictions apply to the availability of these data. Data cleaning, preprocessing, and model development were done in R version 4.1.2. The programming code for R is available on GitHub: https://github.com/bogwel/AGE-Mortality-Prediction.
