# Supplementary material for: A machine learning approach to predicting inpatient mortality among pediatric acute gastroenteritis patients in Kenya
Source: Learn Health Syst. 2024 Dec 26;9(2):e10478. doi: 10.1002/lrh2.10478 (PMC12000769; doi:10.1002/lrh2.10478)
Supplement: Supplementary file 1 — Figure S1. (a) Missingness map for the mortality study for patients aged <5 years admitted with acute gastroenteritis at Siaya County Referral Hospital, western Kenya 2010–2020. (b) Patterns of missing data for the mortality study for patients aged <5 years admitted with acute gastroenteritis at Siaya County Referral Hospital, western Kenya 2010–2020. Figure S2. Business value plots for the champion model predicting in‐hospital mortality among patients aged <5 years admitted with acute gastroenteritis at Siaya County Referral Hospital, western Kenya 2010–2020. Table S1. Trends of mortality among patients aged <5 years admitted at Siaya County Referral Hospital with acute gastroenteritis: 2010–2020. Table S2. Calibration results of mortality prediction models applied to test data. [file LRH2-9-e10478-s001.docx]

**SUPPLEMENTARY APPENDIX**

# **A Machine Learning Approach to Predicting Inpatient Mortality among Pediatric Acute Gastroenteritis Patients in Kenya**

1. **Methods Appendix**
2. **Definition of Key variables**

**Figure S1a.** Missingness map for the mortality study for patients aged < 5 years admitted with acute gastroenteritis at Siaya county referral hospital, western Kenya 2010–2020

**Figure S1b.** Patterns of missing data for the Mortality study for patients aged < 5 years admitted with acute gastroenteritis at Siaya county referral hospital, western Kenya 2010–2020

**Figure S2.** Business value plots for the champion model predicting in-hospital mortality among patients aged < 5 years admitted with acute gastroenteritis at Siaya county referral hospital, western Kenya 2010–2020

**Table S1**. Trends of mortality among patients aged < 5 years admitted at Siaya County Referral Hospital with acute gastroenteritis: 2010-2020

**Table S2.** Calibration results of mortality prediction models applied to test data.

**1.** **Methods Appendix:**

**1.1 Description of Machine Learning Algorithms Used**

LR is a supervised learning classification algorithm that predicts the probability of an outcome variable which is dichotomous in nature [1] while NB is a probabilistic classifier that uses joint probabilities of terms and categories to estimate the probabilities of categories given in a dataset working on the assumption that all terms are conditionally independent of each other in a given category and is insensitive to noise [2]. RF is an algorithm that builds an ensemble of decision trees trained with a bagging approach to get a more accurate and stable prediction[3], GBM - an algorithm that builds strong learners from weak learners in a gradual, additive and sequential manner offering lots of flexibility allowing for optimization of different loss functions [4]. SVM works through discriminating between two classes by generating a hyperplane which optimally segregates classes after the mathematical transformation of input data into a high dimensional space [5]. KNN is a classifier that estimates how likely a data point is to be a member of one group or another based on what group the data points nearest to it are in [6], ANN are computational networks that simulate the human-brain processes thereby establishing empirical relationships between outcome and features [7]. These algorithms have been previously applied in various classification tasks involving disease prediction in healthcare.

**1.2 Boruta Feature Selection**

The minimal, average and maximum Z score of a shadow feature are represented by blue boxplots. Red, yellow and green boxplots represent Z scores of rejected, tentative and confirmed features, respectively. From the Boruta output, features that were either confirmed or tentative were used in model development. A shadow feature is an artificial feature created to help determine the importance of real features. For each real feature in the dataset, a corresponding shadow feature is created by shuffling the values of the real feature so as to maintain the distribution of the data while destroying any relationship with the outcome. By using shadow features, Boruta provides a robust way to assess the significance of real features, effectively distinguishing between noise and informative features.

**1.3 Justification for 75%:25% Data Split**

While there is no established guidelines on data split ratios, it is common practice to use 75%-80% of the total sample for training since a large training set reduces bias and will yield better performance [8].

**1.4 Ensemble Modelling**

Ensemble modelling is the technique where different base learners (level-0 models) are created to predict an outcome and the predictions from these models are then aggregated using a meta-learner (Level-1 model) to make a final prediction [9]. Our stacked ensemble used the following algorithms as base learners: RF, ANN, NB, KNN, GBM, SVM, Linear discriminant analysis (LDA), General additive Models (GAM), and Adaboost. While logistic regression was the algorithm of choice in the meta-learner. A simple algorithm was used for the meta-learner because using a complex meta-learner increases the probability of overfitting the predictions form the base-learners [10].

**1.5 Calibration**

Calibration which involves comparing the model’s prediction against the real (observed) distribution was assessed using Brier scores (the mean squared error between the actual outcome and the estimated probabilities), Spiegelhalter’s *z*-test (a formal measurement that serves as a proxy for calibration calculated from the decomposition of Brier score) and its accompanying p-value [11].

**1.6 Business Value Evaluation**

To evaluate the business value of the predictive model, modelplotr package [12] was used to build valuable evaluation plots (cumulative gains, cumulative lift, response and cumulative response plots). The cumulative gains plot was used to visualize the percentage of the target class members that were selected if we decided to select up until percentile X while the cumulative lift plot was used to explain how much better selecting based on our model was compared to taking random selections. The response plot was used to plot the percentage of target class observations per percentile. Lastly, the cumulative response plot was used to show the expected percentage of the target class observations in the selection, when we apply the model and select up until percentile X.

**1.7 Sample Size Estimation**

Sample size was conducted utilizing a formula developed by Riley et al [13].

$\boldsymbol{n}=\boldsymbol{P}/(\boldsymbol{S}-\mathbf{1})\boldsymbol{ln}(\mathbf{1}-\frac{\boldsymbol{R}^{\mathbf{2}}\boldsymbol{cs}}{\boldsymbol{S}})$ [13]

Where P= Candidate predictor parameters; S= 1- shrinkage ; R2cs- Cox-Snell R squared statistic ;

P was 25 for mortality prediction; desired shrinkage level was ≤ 10% S=0.9 and R2cs is at least 0.1

For mortality prediction:

n=P / ((S-1)ln(1-(R2cs/S)))

n=25/((0.9-1)ln(1-(0.1/0.9)))

n=2,122

The sample size calculation closely aligns with the total population count. Consequently, we opted to include the entire population in the primary studies as participants for each diarrheal outcome in this particular study.

**2. Definition of Variables**

**Wasting:** Weight for Height Z-score (WHZ) were categorized as: normal if > -2.0; Moderate if -3.0 to less than -2.0; Severe if less than -3.0.

**Stunting:** Height for Age Z-score (HAZ) categorized as**:** normal if > -2.0; Moderate if -3.0 to less than -2.0; Severe if less than -3.0.

**Dehydration:** WHO classification used:

- Severe Dehydration: Child exhibiting at least two of the following signs: Lethargic or unconscious; sunken eyes; Drinks poorly or is unable to drink; Skin pinch goes back very slowly.
- Some Dehydration: Child exhibiting at least two of the following signs: Restless or irritable; sunken eyes; drinks eagerly or is very thirsty; skin pinch goes back slowly

**Fever:** Caregiver reported fever/hotness of body.

**Weight-loss:** Caregiver reported loss of weight with the illness

**Vesikari Score**: Severity of diarrheal illness was assessed using Vesikari as described in Table below:


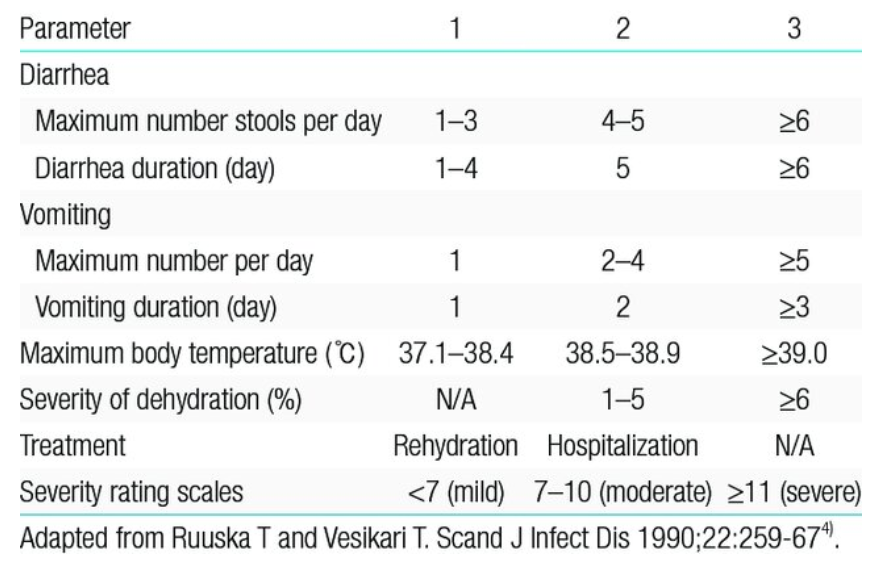


**
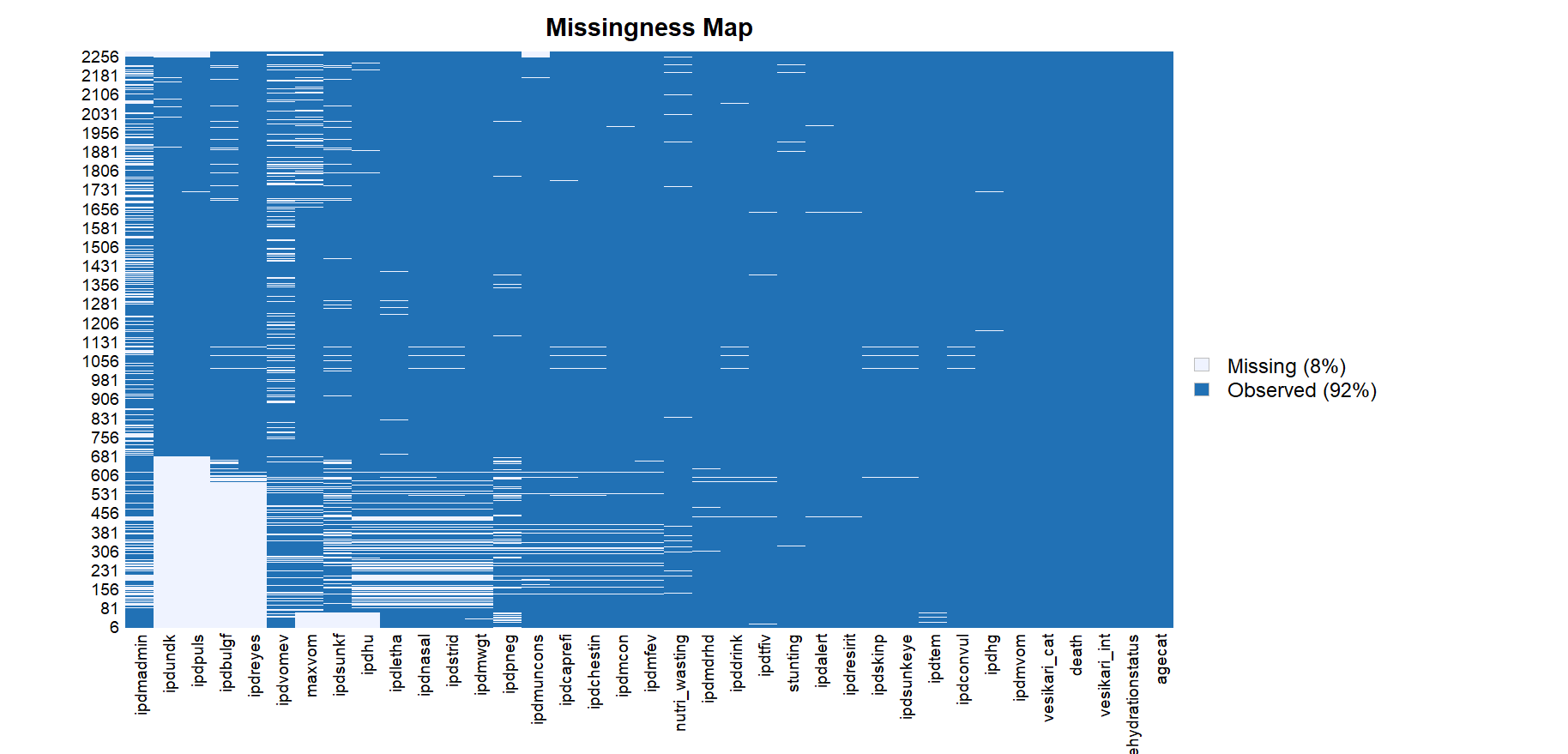
**

**Figure S1a.** Missingness map for the mortality study for patients aged < 5 years admitted with acute gastroenteritis at Siaya county referral hospital, western Kenya 2010–2020


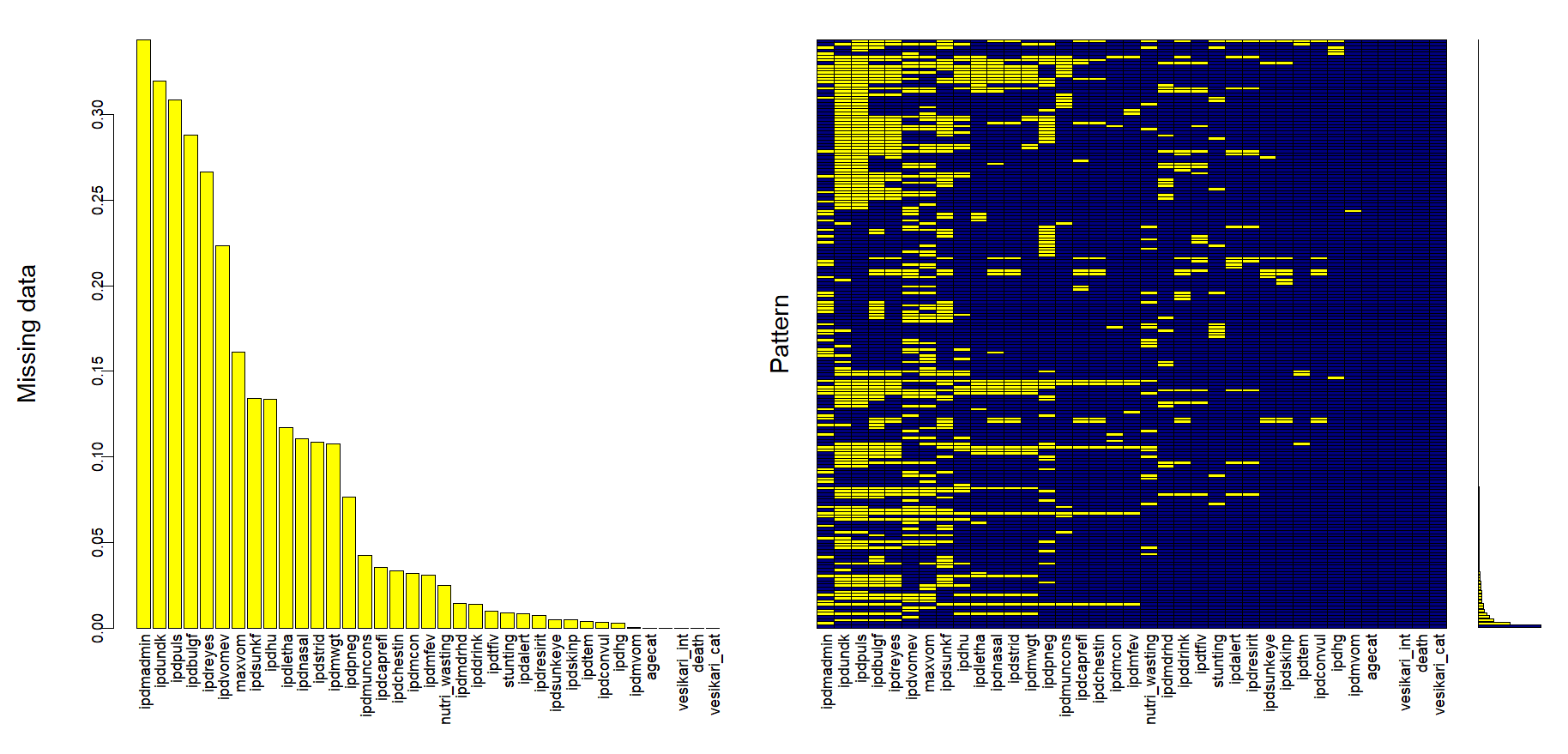


**Figure S1b.** Patterns of Missing Data for the Diarrhea-associated Mortality study for patients aged < 5 years admitted with acute gastroenteritis at Siaya county referral hospital, western Kenya 2010–2020


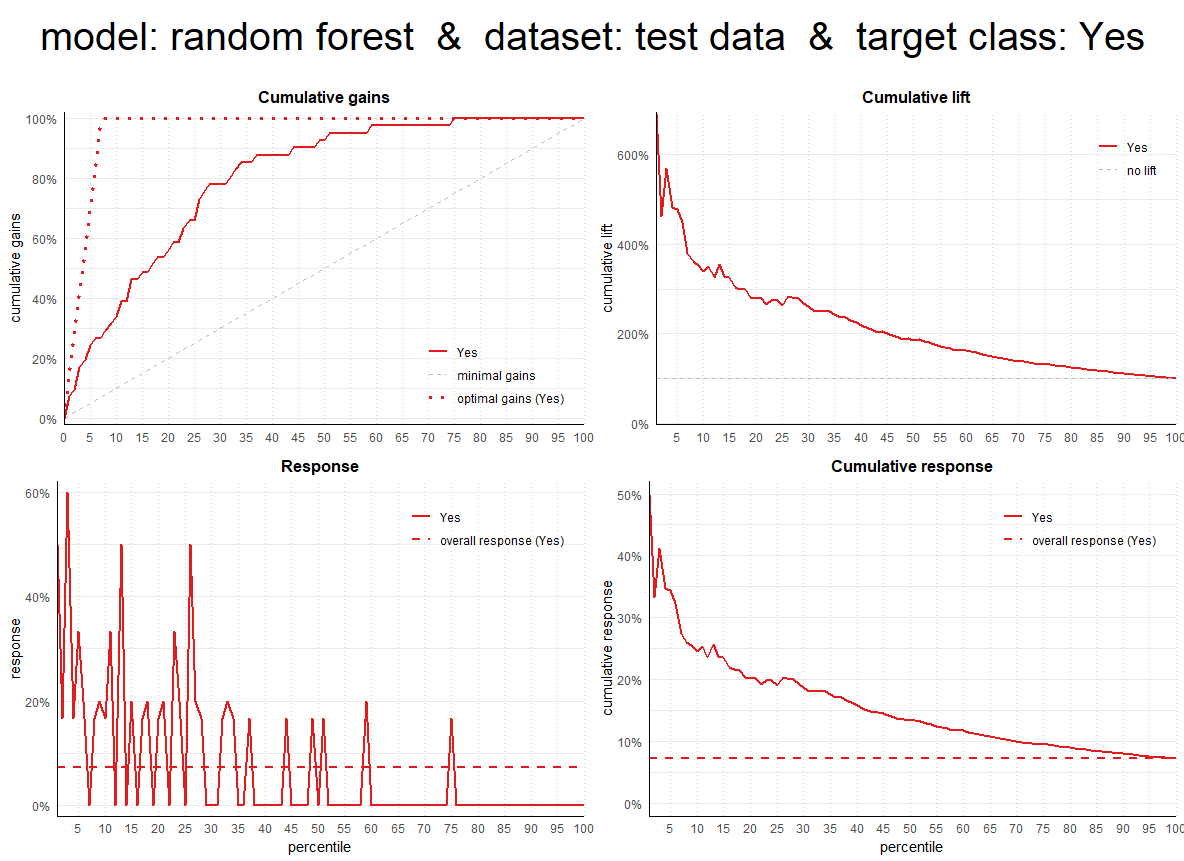


Figure S2. Business value plots for the champion model predicting in-hospital mortality among patients aged < 5 years admitted with acute gastroenteritis at Siaya county referral hospital, western Kenya 2010–2020

**Table S1**. Trends of mortality among patients aged < 5 years admitted at Siaya County Referral Hospital with acute gastroenteritis: 2010-2020

| Year | Death (n=164) | Survived (n=2,107) | Percent | P-value for trend |
| --- | --- | --- | --- | --- |
| 2010 | **40** | **614** | **6.1%** | **0.3142** |
| 2011 | 35 | 452 | 7.2% |  |
| 2012 | 23 | 253 | 8.3% |  |
| 2013 | 24 | 167 | 12.6% |  |
| 2014 | 23 | 209 | 9.9% |  |
| 2015 | 6 | 122 | 4.7% |  |
| 2016 | 5 | 65 | 7.1% |  |
| 2017 | 3 | 34 | 8.1% |  |
| 2018 | 0 | 47 | 0.0% |  |
| 2019 | 1 | 58 | 1.7% |  |
| 2020 | 4 | 86 | 4.4% |  |
| Overall | **164** | **2,107** | 7.2% |  |

**Table S2**. Calibration results of mortality prediction models applied to test data.

| Algorithm | Brier Score | Spiegelhalter Z-score | Spiegelhalter p-value |
| --- | --- | --- | --- |
|  |  |  |  |
| RF | 0.16 | -6.62 | <0.0001 |
| GBM | 0.15 | -1.91 | 0.0558 |
| NB | 0.22 | 19.59 | <0.0001 |
| LR | 0.20 | 3.89 | <0.0001 |
| SVM | 0.20 | -0.17 | 0.863 |
| KNN | 0.22 | 20.29 | <0.0001 |
| ANN | 0.44 | 30.97 | <0.0001 |

*RF-Random Forest; GBM- Gradient Boosting Machine; NB-Naïve Bayes; LR-Logistic Regression; SVM- Support Vector Machine; KNN- K-Nearest Neighbors; ANN- Artificial Neural Networks

**References**

1. Shipe ME, Deppen SA, Farjah F, Grogan EL. Developing prediction models for clinical use using logistic regression: an overview. J Thorac Dis **2019**; 11:S574–S584.

2. Langarizadeh M, Moghbeli F. Applying Naive Bayesian Networks to Disease Prediction: a Systematic Review. Acta Inform Med **2016**; 24:364–369.

3. Khalilia M, Chakraborty S, Popescu M. Predicting disease risks from highly imbalanced data using random forest. BMC Medical Informatics and Decision Making **2011**; 11:51.

4. Zhongheng Zhang YZ. Predictive analytics with gradient boosting in clinical medicine. Annals of Translational Medicine **2019**; 7. Available at: https://www.ncbi.nlm.nih.gov/pmc/articles/PMC6511546/. Accessed 19 October 2021.

5. Yu W, Liu T, Valdez R, Gwinn M, Khoury MJ. Application of support vector machine modeling for prediction of common diseases: the case of diabetes and pre-diabetes. BMC Medical Informatics and Decision Making **2010**; 10:16.

6. Shee H, Cheruiyot W, Kimani S. Application of k-Nearest Neighbour Classification in Medical Data Mining. **2014**; 4.

7. Shahid N, Rappon T, Berta W. Applications of artificial neural networks in health care organizational decision-making: A scoping review. PLOS ONE **2019**; 14:e0212356.

8. Li HL and M. 7.2 Data Splitting and Resampling | Introduction to Data Science. 2022. Available at: http://scientistcafe.com/IDS/. Accessed 27 January 2023.

9. Kotu V, Deshpande B. Chapter 2 - Data Mining Process. In: Kotu V, Deshpande B, eds. Predictive Analytics and Data Mining. Boston: Morgan Kaufmann, 2015: 17–36. Available at: https://www.sciencedirect.com/science/article/pii/B9780128014608000021. Accessed 9 December 2022.

10. Lim Y. Stacked ensembles — improving model performance on a higher level. 2022. Available at: https://towardsdatascience.com/stacked-ensembles-improving-model-performance-on-a-higher-level-99ffc4ea5523. Accessed 27 January 2023.

11. Huang Y, Li W, Macheret F, Gabriel RA, Ohno-Machado L. A tutorial on calibration measurements and calibration models for clinical prediction models. Journal of the American Medical Informatics Association **2020**; 27:621–633.

12. Nagelkerke J. modelplotr: Plots to evaluate the business value of predictive models. 2020; Available at: https://cran.r-project.org/web/packages/modelplotr/vignettes/modelplotr.html. Accessed 19 November 2022.

13. Riley RD, Ensor J, Snell KIE, et al. Calculating the sample size required for developing a clinical prediction model. BMJ **2020**; 368:m441.
